# Improving climate suitability for *Bemisia tabaci* in East Africa is correlated with increased prevalence of whiteflies and cassava diseases

**DOI:** 10.1038/s41598-020-79149-6

**Published:** 2020-12-16

**Authors:** Darren J. Kriticos, Ross E. Darnell, Tania Yonow, Noboru Ota, Robert W. Sutherst, Hazel R. Parry, Habibu Mugerwa, M. N. Maruthi, Susan E. Seal, John Colvin, Sarina Macfadyen, Andrew Kalyebi, Andrew Hulthen, Paul J. De Barro

**Affiliations:** 1 CSIRO, GPO Box 1700, Canberra, 2601 Australia; 2 University of Queensland, Brisbane, QLD 4072 Australia; 3 InSTePP, University of Minnesota, St. Paul, MN 55108 USA; 4 CSIRO, GPO Box 2583, Brisbane, QLD 4001 Australia; 5 CSIRO, Private Bag 5, Wembley, WA 6913 Australia; 6 Root Crops Programme, National Crops Resources Research Institute, P. O. Box 7084, Kampala, Uganda; 7 Natural Resources Institute (NRI), University of Greenwich, Chatham Maritime, Kent ME4 4TB UK

**Keywords:** Ecological modelling, Ecological epidemiology, Entomology, Agroecology, Climate-change ecology, Ecological modelling, Invasive species, Population dynamics

## Abstract

Projected climate changes are thought to promote emerging infectious diseases, though to date, evidence linking climate changes and such diseases in plants has not been available. Cassava is perhaps the most important crop in Africa for smallholder farmers. Since the late 1990’s there have been reports from East and Central Africa of pandemics of begomoviruses in cassava linked to high abundances of whitefly species within the *Bemisia tabaci* complex. We used CLIMEX, a process-oriented climatic niche model, to explore if this pandemic was linked to recent historical climatic changes. The climatic niche model was corroborated with independent observed field abundance of *B. tabaci* in Uganda over a 13-year time-series, and with the probability of occurrence of *B. tabaci* over 2 years across the African study area. Throughout a 39-year climate time-series spanning the period during which the pandemics emerged, the modelled climatic conditions for *B. tabaci* improved significantly in the areas where the pandemics had been reported and were constant or decreased elsewhere. This is the first reported case where observed historical climate changes have been attributed to the increase in abundance of an insect pest, contributing to a crop disease pandemic.

## Introduction

Projected changes to climate are widely believed to affect the range and abundance patterns of many arthropod pests, weeds and diseases^1–7^. Whilst changes in abundance and voltinism within species ranges have been posited in relation to expected climatic changes^8,9^, detecting such changes has been challenging^10–13^. Climatic systems are typically complicated and noisy across broad ranges of spatial and temporal scales, making it difficult to discern trends against the background variation in climatic variables^14,15^. Detecting trends in biological phenomena that are influenced by climate is an even greater challenge due to the added layers of system complexity^16–18^. One such system type that is theoretically sensitive to climate change is emerging infectious diseases that are transmitted by insects^19^.

Cassava, *Manihot esculenta* Crantz (Euphorbiaceae) is one of the most important subsistence crops in Africa, with the highest total production in terms of fresh weight; almost 158 million tonnes produced in 2013^20^; and is the second most important source of dietary energy after maize^21^. Cassava has a critical role in assuring long-term food security^22^. The plants drought tolerance and hardiness^23^ have underpinned hopes this crop can play a significant role in climate adaptation, providing sustenance and income to low-income farmers in particular^24,25^. Pandemics of cassava mosaic disease (CMD) and cassava brown streak disease (CBSD) have been reported widely throughout East and Central Africa since the late 1990′s^26–33^. The cassava production losses to CMD and CBSD of up to 47% in East and Central Africa^34^ have raised widespread concerns regarding food security over the past decade^35^.

A number of hypotheses have been propounded to explain the present cassava disease epidemics in East and Central Africa, including the development of a novel recombinant begomovirus causing severe CMD^36^, the range expansion of a native ‘invader’ *Bemisia tabaci* (Gennadius) (Hemiptera: Aleyrodidae) (Ug2) whitefly vector population^37^, the synergistic interaction between the high *B. tabaci* population and the viruses that cause severe disease^27^, and changes in genetics of *B. tabaci*^30^, possibly through hybrid introgression^28^. Garrett, et al.^38^ hints at the possibility that climate change may be a causal factor in the emergence of cassava diseases in East and Central Africa but stops short of examining the question directly.

*Bemisia tabaci* is a cryptic species complex^39^ consisting of highly damaging pests of agriculture, damaging crops through both feeding and transmission of a range of plant viruses, including begomoviruses and ipomoviruses that cause CMD and CBSD, respectively^28^. For over 20 years, *B. tabaci* have been reported to be increasing in abundance on cassava in East and Central Africa^27,28^. There are many factors influencing high abundance of *B. tabaci* in cassava fields of East Africa^40^; though few studies address the impact of long-term climate patterns^41^.

Any discussion of *B. tabaci* requires at least some consideration of its taxonomy^42,43^*.* A recent taxonomic treatment identifies that *B. tabaci* consists of at least 39 morphologically indistinguishable species^44^, though Tay, et al.^39^ revealed that pseudogenes were present in *Bemisia,* conflating the apparent genetic diversity in previous treatments. The most impactful species in the complex globally has been termed Middle East-Asia Minor 1 (MEAM1), formerly termed biotype B^45,46^. It has not however been recorded as a pest of cassava. In East and Central Africa, the most common *B. tabaci* species on cassava have been identified as belonging to the Sub-Saharan African taxa (SSA1, SSA2, and SSA3)^28,30,43,45^ with SSA1 the most prevalent species in the region^47^. Within SSA1 *B. tabaci*, two subgroups (SG) designated as SG1 and SG2 based on their > 1.3% nt divergence in partial mtCO1 sequences^28^ are referenced in this study. While we do not advocate for the use of subgroups as a taxonomic classification, we use them here for consistent reference to previous work. In addition, SSA2 is the other SSA *B*. *tabaci* used in this study and diverges from SSA1 subgroups by > 8% nt in partial mtCO1 sequences^28^.

Simple correlative species distribution modelling techniques have been used to estimate the potential distribution of *B. tabaci* s.l. and other high-profile risk factors for cassava production^48^. These descriptive consensus models provide little if any insight into the climatic factors that limit or promote the presence of *B. tabaci* s.l. or the plant viruses it spreads. These models are generally poorly suited for extrapolation in space or time^49^, or for exploring the abundance patterns of invasive organisms^50^. The correlative modelling results in Herrera Campo, et al.^48^ are of poor and erratic quality; they have relatively low sensitivity, and provide projections into implausibly cold, dry and hot-wet habitats.

A more recent use of a correlative species distribution model attempted to explore climate change implications on the potential distribution of *B. tabaci*, Biotype B and Q (syn *B. tabaci* MEAM1 and MED respectively)^51^. The baseline model does not fit the distribution training data very well, with significant omission in the USA (north of Florida), parts of Africa (North Sudan and western Tanzania) and throughout equatorial parts of South-Eastern Asia (Indonesia and the Philippines). There also appear to be modelling artefacts in the results for South America, Africa and Australia, with a patchwork pattern of modelled suitability suggesting model over-fitting or the use of inappropriate covariates. The modelled unsuitability in Angola, Zambia, Botswana and Namibia is doubtful given the climatic similarity to regions such as northeastern Australia where *B. tabaci* has been recorded frequently. The inclusion of the northern Chinese records in the training dataset suggests a misunderstanding of the difference between “field-caught records” and evidence of establishment. In this case, the records are likely populations that over-wintered in glasshouses and were then subsequently caught in the field. Curiously, the inclusion of these northern Chinese records stretched the MaxEnt model to include these regions as climatically suitable, but it did not result in areas elsewhere with similar climates being identified as similarly suitable (i.e., central western USA). These features and the poor fit to the training data does not inspire confidence in this model. In the face of such deficiencies, the relatively high AUC scores (~ 0.89) underscores concerns regarding the use of this statistic to evaluate bioclimatic models^52^. The application of climate change scenarios to correlative species distribution models is an inherently unreliable method because it requires extrapolation into novel climates defined in terms of an n-dimensional space typically consisting of bioclim variables^49^. The future climate model scenarios in Ramos et al.^51^ reinforce this caution, with implausible, noisy modelling artefacts in the results.

CLIMEX is an ecoclimatic modelling package for exploring the effects of climate on plants, animals and diseases^53,54^. CLIMEX has been used to assess the potential distribution of a broad range of invasive organisms such as plant pathogens^55,56^, insects^57–60^ and plants^1,49^, and to simulate the seasonal dynamics of pest species (e.g.,^61^) and even long term changes^62,63^. CLIMEX models benefit from the modeller being able to cross-validate parameters and results across three different knowledge domains: distribution data, phenological observations, and experimental observations of development and survival under laboratory or field conditions. This cross-validation process typically reveals errors and interpretation issues in distribution data (e.g., geocoding errors and the effects of human climate-modifying habitat factors such as irrigation and glasshouses) and helps ensure that included model components and parameters are biologically plausible. A new development in CLIMEX Version 4 is the ability to run the Compare Locations model on a monthly time series climate database, providing the first opportunity to simulate and visualise the seasonal and inter-annual spatio-temporal patterns of climate suitability.

**Figure 1:**
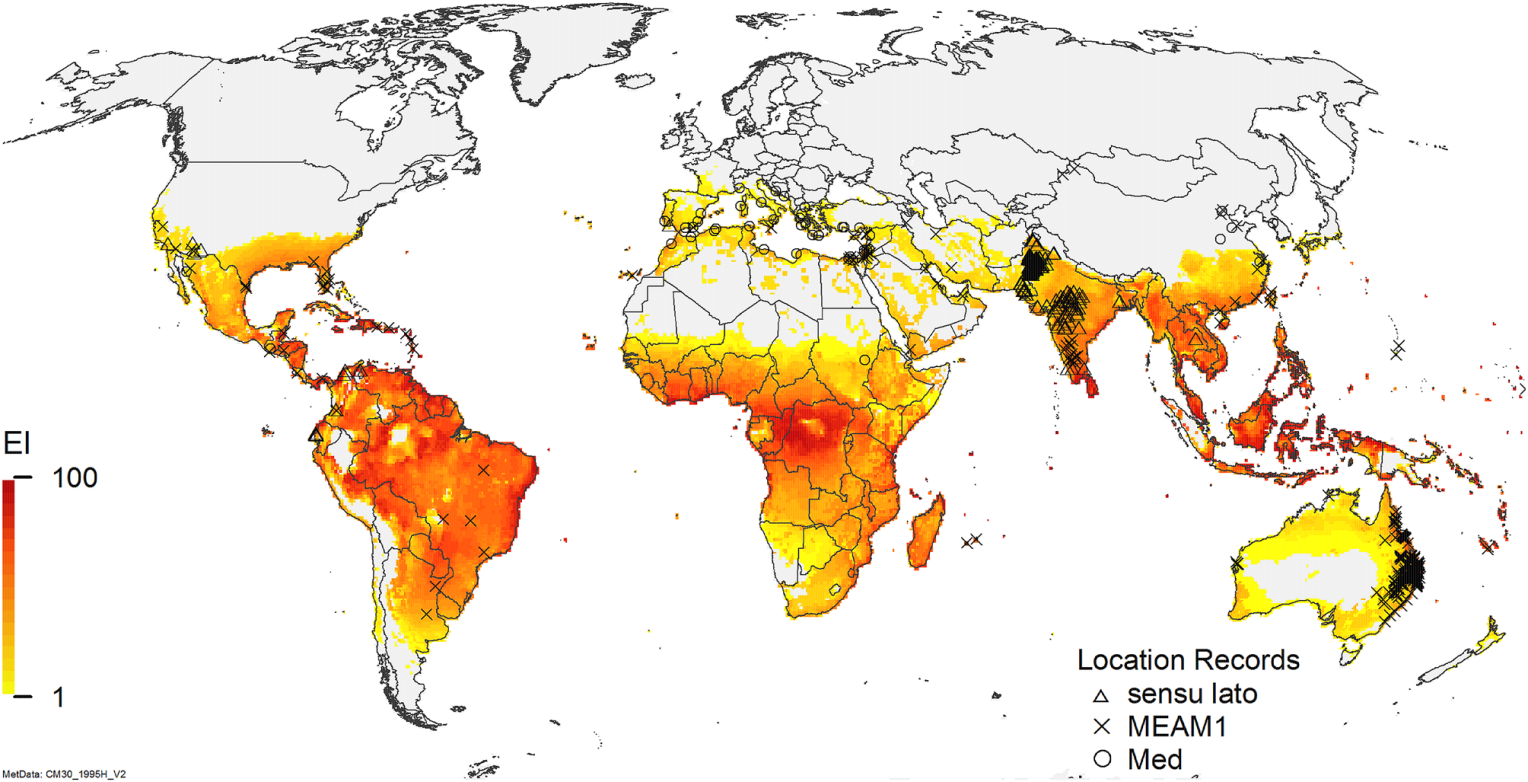
Modelled global potential distribution of *Bemisia tabaci* s.l. under a composite scenario of natural rainfall and irrigation (Kriticos et al.^64^). Outlying location records in northern China are thought to represent populations that overwinter in glasshouses in areas that are otherwise unsuitably cold for population persistence of *B. tabaci* MEAM1. The climate data is the CM30 1995H V2 dataset^65^. Map produced using ArcMap 10.6 (ESRI, Redlands, Ca., esri.com).

**Figure 2:**
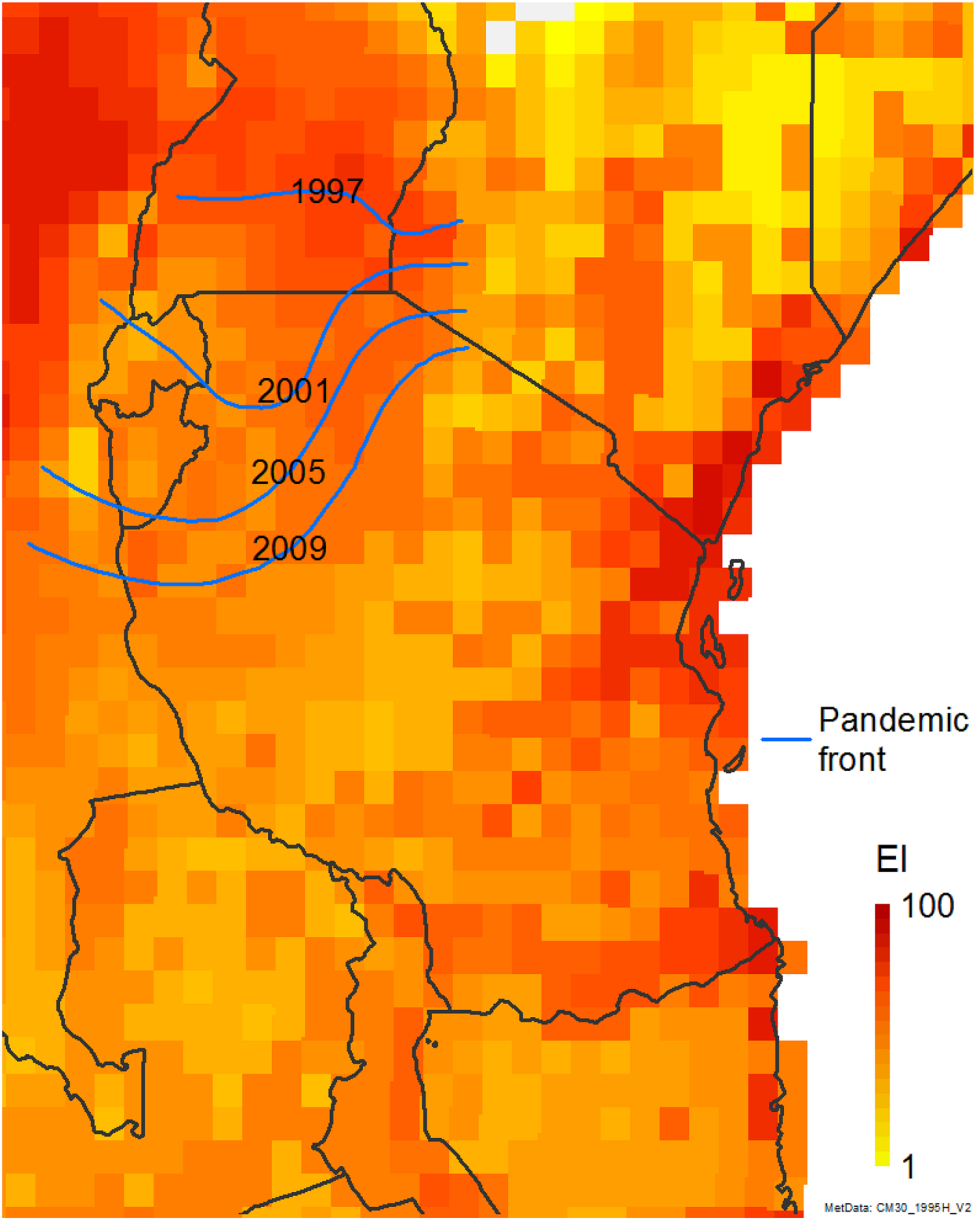
Modelled climate suitability of *Bemisia tabaci* s.l. across the study area in East Africa under a composite scenario of natural rainfall and irrigation (Kriticos et al.^64^). The climate data is the CM30 1995H V2 dataset^65^. Pandemic fronts digitised from Legg, et al.^28^. Map produced using ArcMap 10.6 (ESRI, Redlands, Ca., esri.com).

A CLIMEX model has been developed for *B. tabaci* MEAM1 (Fig. 1)^64^. This model was fitted to describe the relative climate suitability for *B. tabaci* MEAM1 using long-term climate averages from 1981 to 2010, centred on 1995^65^. The resulting climate suitability model indicated that the potential distribution of *B. tabaci* MEAM1 included all of the known distribution records for all of the members of the *B. tabaci* complex, including the known distribution of the SSA species and the area within which the cassava disease epidemics had been observed (Fig. 2). There is a large degree of similarity in ecophysiological parameters between different species within the *B.* *tabaci* complex (e.g. temperature-dependent development rates)^64^. The geographical distribution of the different species within the complex includes regions of range overlap (e.g., MED and MEAM1 in northern China, and SSA taxa in Eastern and Central Africa), and also more discrete clustering. This suggests that the fundamental niche of these cryptic species may have a large degree of overlap, and their realised niches reflect differences in biotic factors such as the distribution of hosts and natural enemies, interspecific competition and interactions with plant diseases^66–68^. The range boundaries for a given taxa tend to reflect spatial demographic processes, and competitive advantage can be conferred by even slight differences in ecophysiological performance^69,70^.

In this paper, we apply the ‘end-to-end’ method of Rosenzweig et al.^18^ to explore the role of historical climatic changes in the cassava disease epidemic in parts of sub-Saharan Africa. To confirm that the CLIMEX model fitted to *B. tabaci* MEAM1 is relevant to the SSA *B. tabaci* we compare the temperature development response patterns for species of *B. tabaci* found associated with the cassava disease outbreaks in sub-Saharan Africa with those observed for MEAM1 and MED. We run the *B. tabaci* MEAM1 CLIMEX model of Kriticos et al.^64^ with the CLIMEX Compare Locations/Years module, using a set of gridded monthly climate time series to test the hypothesis that the modelled annual climate suitability for *B. tabaci* MEAM1 is correlated with a 13-year time series of abundance of *B. tabaci* in Uganda^71^, and that the correlation is stable through time. As a further check of model relevance, we compare the CLIMEX model outputs for long-term average climate with data on *B. tabaci* abundance collected from cassava fields across Uganda, Tanzania and Malawi^72^. Finally, we use the time series of climate suitability to test whether the climate suitability for *B. tabaci* in East and Central Africa has been increasing when and where *B. tabaci* SSA, CMD and CBSD have been observed to increase in prevalence^28,30^. In so doing, we explore whether we can attribute the observed epidemiological and ecological changes to changes in modelled climatic suitability for *B. tabaci* SSA species.

## Methods

### CLIMEX

The CLIMEX Compare Locations model simulates a species population response to climate on a weekly timescale; tracking the potential for population growth during favourable seasons, and population decreases during the stressful seasons. CLIMEX calculates an annual Growth Index (GI_A_) to describe the potential for population growth as a function of weekly soil moisture and temperature during favourable conditions (Eq. 1).

1 $${\text{GI}}_{{\text{A}}} = 100\sum\limits_{i = 1}^{52} {GI_{{W_{i} }} } /52$$

where GI_W_ is the weekly Growth Index, composed of the weekly Temperature Index multiplied by the weekly Moisture Index. The Temperature and Moisture Indices are specified with a functional form that accords with the Law of Tolerance^73–75^, and are combined in a multiplicative manner that accords with the Law of the Minimum^76^. CLIMEX employs up to four stress indices (cold, wet, hot, dry) and their interactions (cold–wet, cold–dry, hot–wet and hot–dry); respectively *CS, WS, HS, DS, CWX, CDX, HWX, HDX*) to estimate the ability of the population to survive unfavourable conditions (Eqs. 2, 3).

2 $$SI \, = \, \left( {1 - \frac{CS}{{100}}} \right)\left( {1 - \frac{DS}{{100}}} \right)\left( {1 - \frac{HS}{{100}}} \right)\left( {1 - \frac{WS}{{100}}} \right)$$

3 $$SX \, = \, \left( {1 - \frac{CDX}{{100}}} \right)\left( {1 - \frac{CWX}{{100}}} \right)\left( {1 - \frac{HDX}{{100}}} \right)\left( {1 - \frac{HWX}{{100}}} \right)$$

The interaction stresses are used infrequently, and often only one of these is employed in any single model.

The Ecoclimatic Index (EI) summarises the balance between the opportunity for the species to grow during the favourable season(s) and the requirement to survive inclement season(s), and scales from 0 (unsuitable) to 100 (optimal) (Eq. 4).

4 $$EI \, = \, GI_{A} \times SI \times SX$$

Very large values of EI are unusual and are restricted to environments that are climatically highly stable, e.g. the wet tropics.

For the Compare Locations model a 30-year average of monthly climate data from 1981 to 2010 was derived from the time series dataset. We calculated the 30-year average from the abovementioned minimum and maximum temperature and precipitation. We then calculated relative humidity at 9:00 and 15:00 h by (1) calculating saturated vapour pressure at these times using the Magnus equation (Eqs. 4, 5 and 6, Kriticos et al.^65^); (2) obtaining relative humidity by dividing vapour pressure by saturated vapour pressure (Eq. 1, Kriticos et al.^65^); and (3) averaging 30-years by month. The minimum and maximum temperature, precipitation, and relative humidity were collated into an Sqlite database for use in CLIMEX Compare Locations.

The Compare Locations/Years model was run with a 39-year monthly time-series climate data provided by the University of East Anglia’s Climatic Research Unit (CRU) data^77,78^. Climate data with a spatial resolution of 0.5° × 0.5° were extracted for monthly averages of daily maximum temperature, maximum temperature, vapour pressure and monthly totals for precipitation.

### Comparing development rates of Sub-Saharan African *Bemisia tabaci* with MEAM1

In order to assess whether the CLIMEX model parameters fitted for *B. tabaci* MEAM1 were relevant for modelling the climate suitability for SSA *B. tabaci*, we assessed the development rates of three *B. tabaci* SSA taxa [identified as SSA1-SG1, SSA1-SG2 and SSA2 based on their partial mtCO1 sequences in line with Legg et al.^28^ subgroupings] as a function of temperature under laboratory conditions. Until recently, the known geographical range of SSA taxa was scant due to limited molecular biological assays of *B. tabaci* in Sub-Saharan Africa^45,46^. Furthermore, their distribution is likely to be complicated by inter-specific competition from within the *B. tabaci* complex, and the importance of crop hosts as a likely range-discriminating factor between these species^43,79^. A consequence of this is that we are unable to reliably infer any stress parameters for these taxa based on their geographical distribution. On the other hand, the soil moisture parameters are strongly associated with the hosts, and factors such as the minimum soil moisture for growth are closely associated with the permanent wilting point, which does not vary much between species. The SSA1-SG1 and SSA1-SG2 *B. tabaci* colonies used in this study were established from *B. tabaci* collected from Kayingo, Uganda in February 2016. The SSA2 *B. tabaci* colony used in this study was established from *B. tabaci* collected from Kiboga, Uganda in August 2013.

Six temperature treatments: 15, 20, 25, 30, 35 and 40 °C which were constant for day and night were used in the experiment. Each treatment was replicated five times across five cassava plants of cultivar Ebwanateraka, each in a Lock and Lock (LL) container. In some cases, one or two replicates out of the five failed, and hence reliable data was only obtained for three or four replicates for such treatments. The cassava cultivar Ebwanateraka was used because it was the predominant variety grown by farmers before the cassava mosaic disease pandemic in the early 1990s^80^.

Ten pairs (10 female and 10 male whiteflies) were introduced onto a cassava plant in an LL container. The LL container with the introduced whiteflies was left at room temperature (25 °C) for 24 h in the insectary at 14:10 light and darkness, at a relative humidity of 60%. After 24 h of feeding and ovipositing, all the adult whiteflies were removed from the cassava plant in the LL container and the number of eggs laid was recorded. Cassava plants in the LL containers were maintained at room temperature in the insectary for another 9 days and on the tenth day the numbers of emerged nymphs on each leaf were recorded. After recording the number of emerged nymphs, plants in the LL containers were transferred to A1000 growth chambers (Conviron Europe limited, UK) set at a given temperature treatment, 14:10 light and darkness, relative humidity of 60% for 5 days. On day 16, cassava plants in the LL containers were removed from the A1000 growth chambers and maintained at room temperature 25 °C in the insectary. Cassava plants in the LL containers were monitored every 2 days for emerged adults. The number of emerged male and female progenies were recorded, and the emerged whiteflies removed from the plant.

Insect development rate was calculated as the inverse of the time taken for the insect to complete development. The mean time taken for whitefly adult emergence (3‒5 replicates) was plotted against temperature for each whitefly taxa, and the response patterns were assessed.

### Climate suitability for *Bemisia tabaci* MEAM1 through time

The CLIMEX Compare Locations/Years model^53^ was run on a monthly time series climate database (Climate Research Unit of the University of East Anglia) for 1978–2017 to simulate and visualise the seasonal and inter-annual spatio-temporal patterns of climate suitability for *B. tabaci* MEAM1^64^. We used the R statistics package^81^ to fit linear time trend models at each spatial grid point to estimate the average annual change in EI, GI_A_, TI and MI across the 39 years in the series. At each point, standardised effect sizes (t value) for this annual change were plotted spatially, as were the 95% lower and upper confidence intervals for the annual change.

### Comparing modelled climate suitability for *Bemisia tabaci* MEAM1 with field abundance of *Bemisia tabaci* in Uganda, 2004–2017

A comprehensive set of data on the field abundance of *B. tabaci* and Cassava Brown Streak Disease (CBSD) in Uganda was published recently^71^. This dataset includes field survey data collected from 2004 to 2017 with a gap for 2016, with 7 627 field summaries across 96 districts. While the survey effort changed in intensity and spatial pattern from year to year, it remains the most useful dataset of its kind. The field data show a clear pattern of invasion of CBSD into cassava fields in Uganda through this period, and these invasion dynamics confound any relationship between prevalence of CBSD and climate suitability for *B. tabaci* sensu lato.

To test the relationship between observed abundance of *B. tabaci* (field counts) and modelled climate suitability for *B. tabaci* MEAM1 throughout the same period as the dataset of Alicai, et al.^71^ we firstly aggregated the field counts to the grid of 121 climate cells covering Uganda used for the CLIMEX modelling. A linear mixed effects model fit by REML was used to model the relationship between modelled EI values in each cell and log (count + 1) for *B. tabaci* in cassava fields and to check for consistency in this relationship through time.

### Comparing modelled climate suitability for ***Bemisia tabaci*** MEAM1 with field abundance of ***Bemisia tabaci*** SSA in 2015 and 2016

In order to further assess if the CLIMEX model fitted to *B. tabaci* MEAM1 could be of use for understanding *B. tabaci* SSA climate suitability, we analysed the relationship between the EI values and observed *B. tabaci* abundance data collected from cassava fields in East and southern Africa. Cassava fields were surveyed in seven regions across Uganda, Tanzania, and Malawi (Supplementary Fig. S1). The first survey took place in September 2015 and the second survey took place in April 2016. In each region, we selected up to 10 fields of a known cassava variety to survey. Categories of adult *B. tabaci* abundance on the top five leaves of 30 plants per field were recorded (0, 1–9, 10–99, 100–200, > 200). For this analysis, only the presence or absence of *B. tabaci* adults was used. *Bemisia tabaci* adults collected here were presumed to be *B. tabaci* SSA (with samples collected to later confirm identity using molecular techniques).

The geocoded locations for the cassava fields were spatially intersected with CLIMEX results for East Africa. A logistic model was fitted in R (using the glm function with a binomial link) to the probability of presence of one or more whiteflies on a cassava plant as a function of CLIMEX EI. We ran the CLIMEX model using a 30-year climate average dataset centred on 1995 using the CRU data described above. The intent in comparing the modelled climate suitability with abundance data was to discern the patterns of agreement, rather than to be able to use modelled climate suitability to predict abundance with any great precision.

## Results

### Development rates

**Figure 3:**
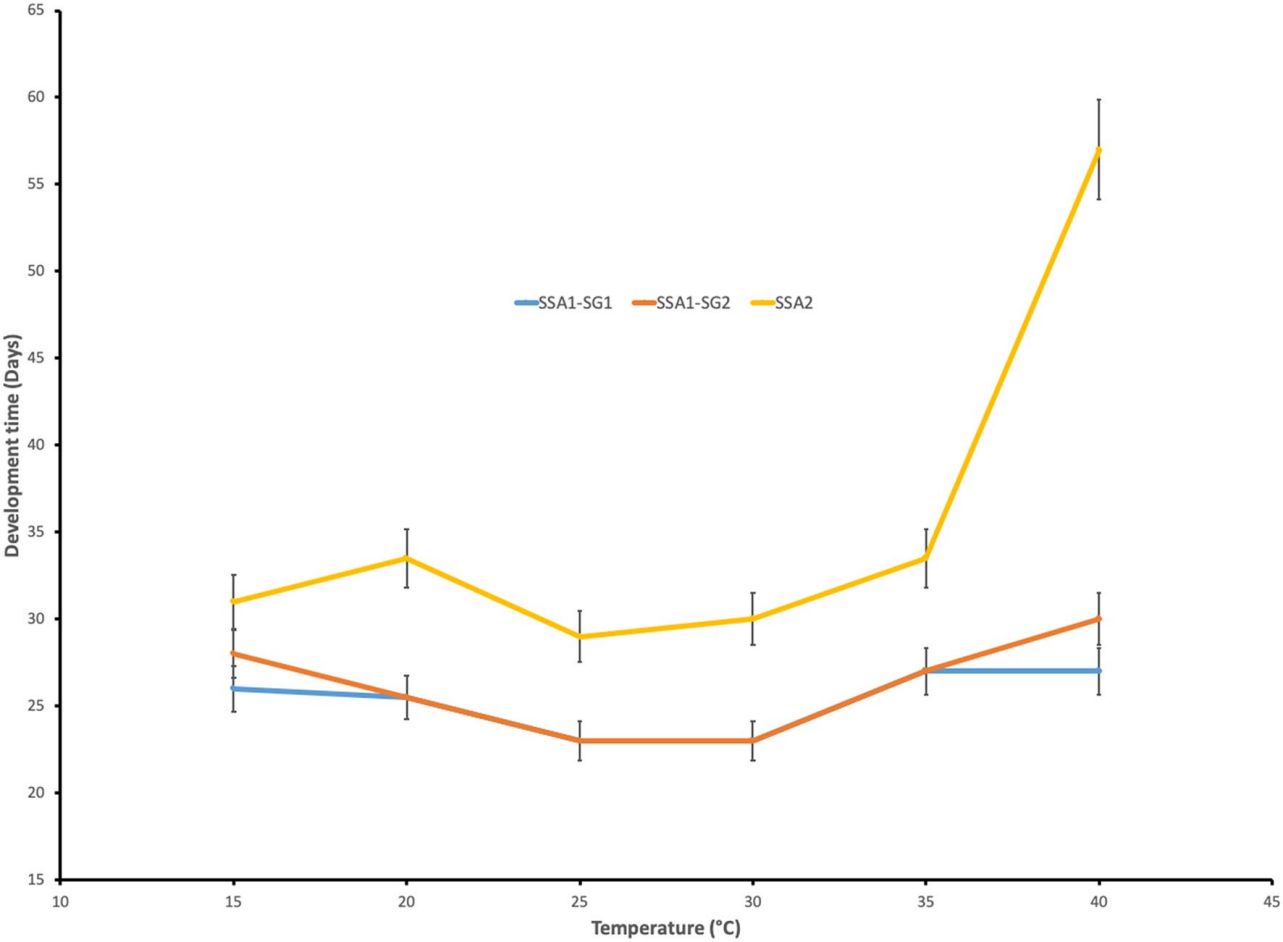
Mean time to develop (± SE) from egg to adult as a function of temperature for each of three *Bemisia tabaci* SSA isofemale lines originally collected in Uganda in 2013 or 2016. Taxonomy follows Legg et al.^28^.

The observed pattern of development rates for *B. tabaci* SSA taxa (Fig. 3) compares favourably with the cardinal temperatures employed in the CLIMEX model originally developed for *B. tabaci* MEAM1^64^:

DV0—minimum temperature for development 12 °C

DV1—lower optimum temperature for development 28 °C

DV2—upper optimum temperature for development 32 °C

DV3—maximum temperature for development 42 °C.

The shortest development time for all taxa occurred between 25 and 30 °C, but the maximum temperature for optimum development was left at 32 °C because we are modelling the suite of SSA *B. tabaci* taxa and one of them displayed optimum development at 30 °C, and the next higher temperature was 35 °C. The maximum temperature for development was set at 42 °C, which is marginally above the maximum experimental temperatures, at which all four SSA taxa were capable of completing development. The development time did increase dramatically after 35 °C, especially for *B. tabaci* SSA2. *Bemisia tabaci* SSA1-SG1 and *B. tabaci* SSA1-SG2 each had similar, and relatively short, development times across all the temperatures assessed. The soil moisture response parameters mostly reflect the niche requirements for crop hosts, with a wet stress consideration in relation to the sensitivity of *B. tabaci* to rainfall^82^. We can therefore feel confident in using the previously developed CLIMEX model to adequately simulate climate suitability for the SSA taxa.

Globally, the potential distribution for *B. tabaci* MEAM1 extends throughout the tropics, sub-tropics, and warm temperate climates, with marginal suitability in the Mediterranean region (Fig. 1). Location records in temperate locations highlight its potential for recurrent invasion from glasshouses, which allow overwintering. The climate suitability for *B. tabaci* MEAM1 in eastern Africa modelled using long-term average climate is quite high in the north west (Uganda) and South-East (coastal Tanzania) of the study area, with a moderate depression in Central Tanzania (Fig. 2)^64^. The modelled climate suitability for *B. tabaci* MEAM1 accords with the distribution records for all of the sub-Saharan African species (sensu^45^) within the *B. tabaci* species complex reported in Legg et al.^28^.

### Field Abundance of ***Bemisia tabaci*** and modelled climate suitability Uganda 2004–2017

**Figure 4:**
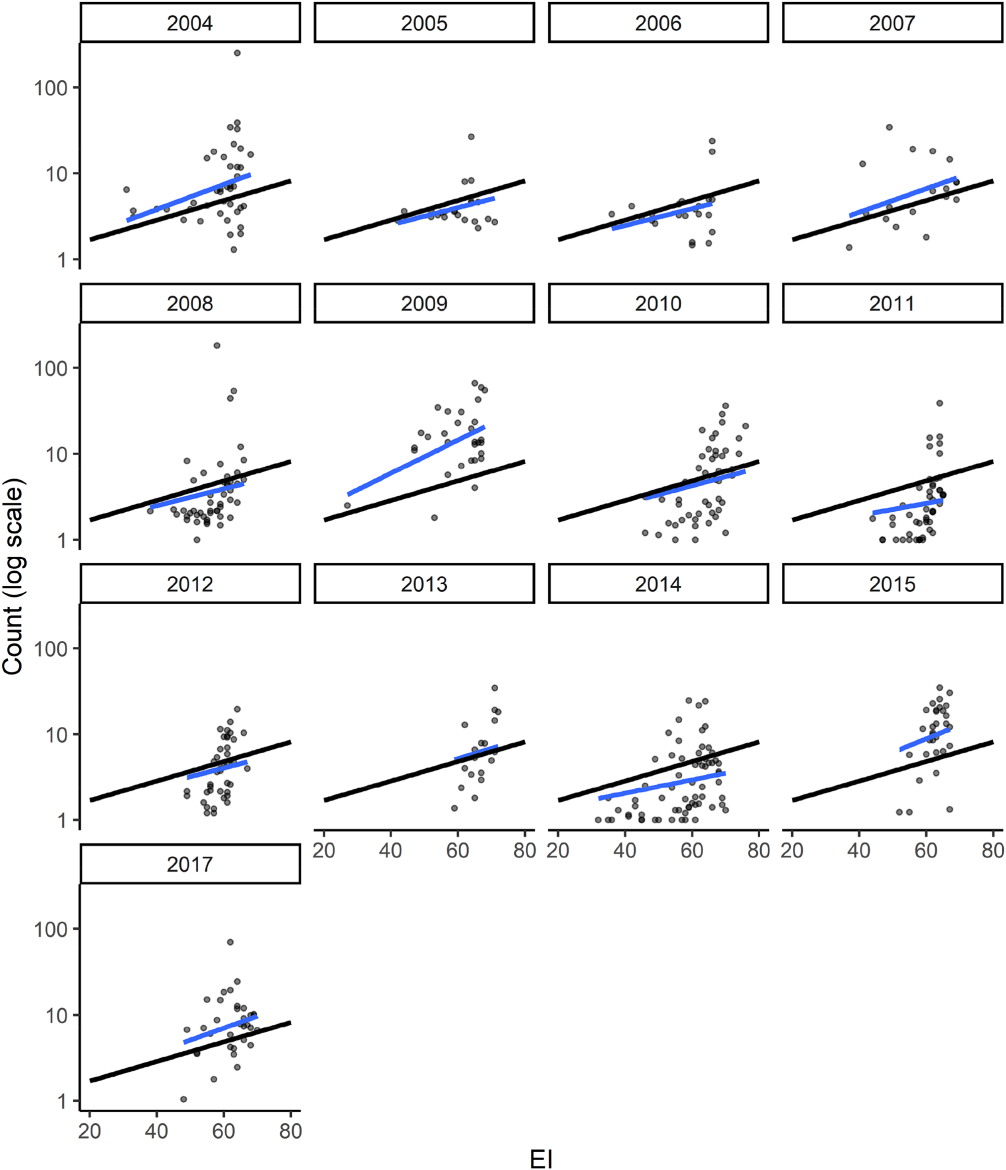
Observed median field abundance of adult *Bemisia tabaci* (species not determined) presence on a cassava plant^71^ as a function of modelled CLIMEX Ecoclimatic Index (EI) for *Bemisia tabaci* MEAM1^64^. Black line represents slope fitted across all years and blue line represents slope fitted to individual years’ data.

The relationship between observed field abundance of *B. tabaci* in Uganda^71^ and modelled climate suitability for *B. tabaci* MEAM1^64^ was statistically consistent throughout the 13 years of surveys, with the variation in slope between years appearing random, with 2009 a standout (Fig. 4).

### Probability of presence of Bemisia tabaci in East Africa 2015–2016

**Figure 5:**
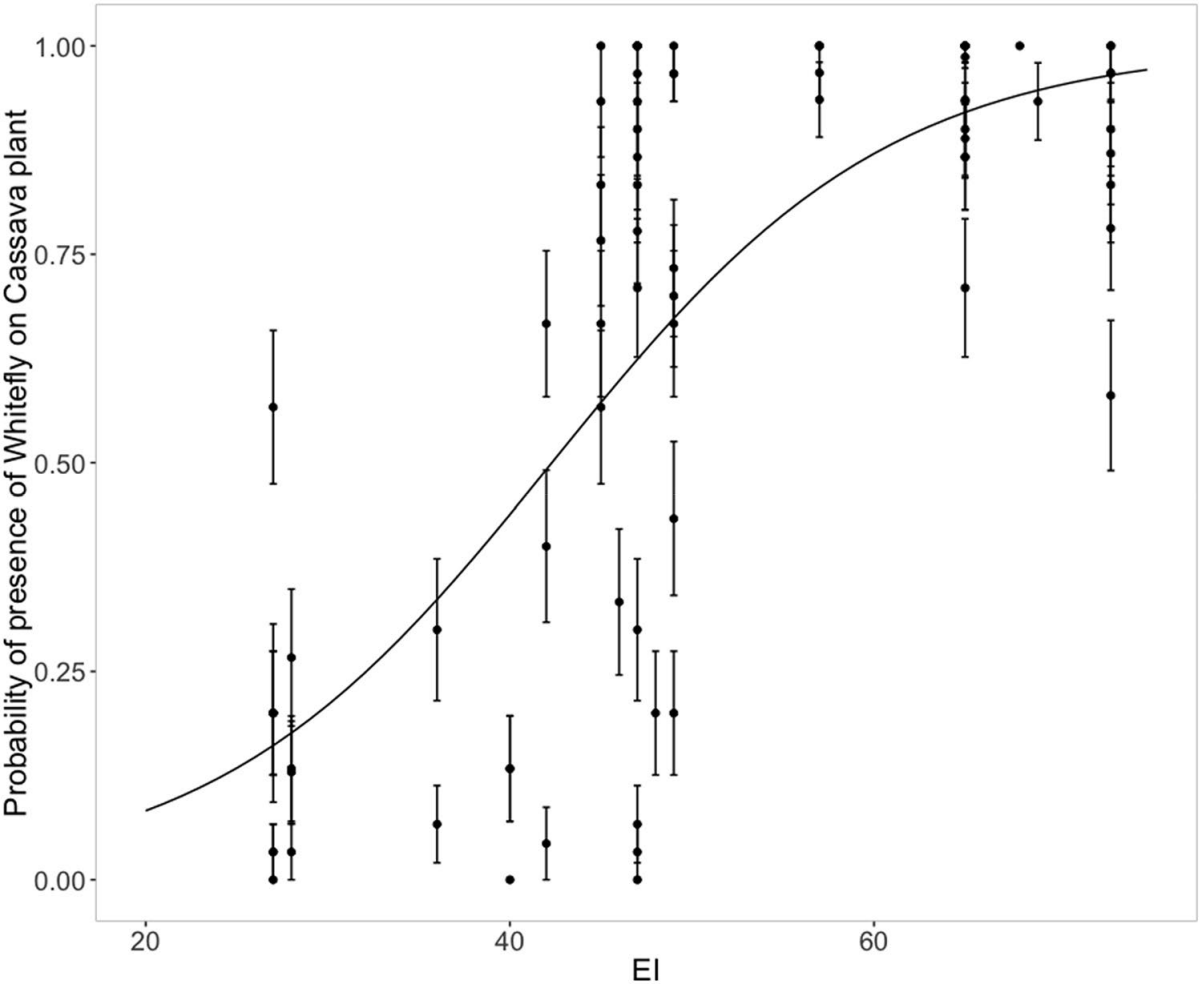
Probability of adult *Bemisia tabaci* (species not determined) presence on a cassava plant as a function of CLIMEX Ecoclimatic Index (EI) for *Bemisia tabaci* MEAM1.

The field abundance patterns (probability of presence of *B. tabaci* on a cassava plant) displays a sigmoid pattern with respect to modelled EI at each site (Fig. 5). There were pronounced differences in prevalence of *B. tabaci* SSA adults between sites at moderate EI values, perhaps reflecting non-climatic influences on population abundance of *B. tabaci* such as crop type, age of the cassava, crop types used across each landscape, natural enemies, competition with other herbivores, and insecticide use.

### Time series

**Figure 6:**
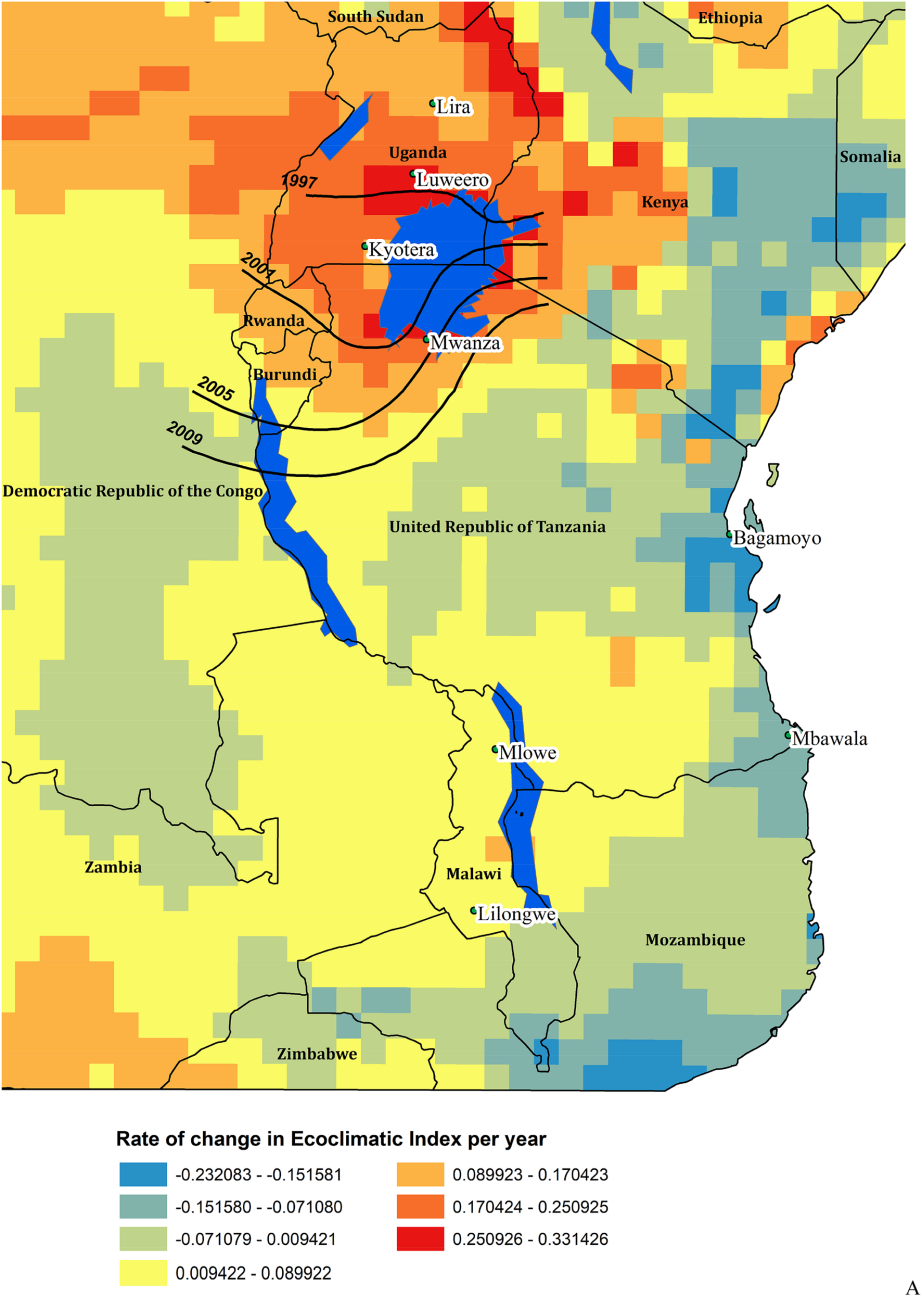

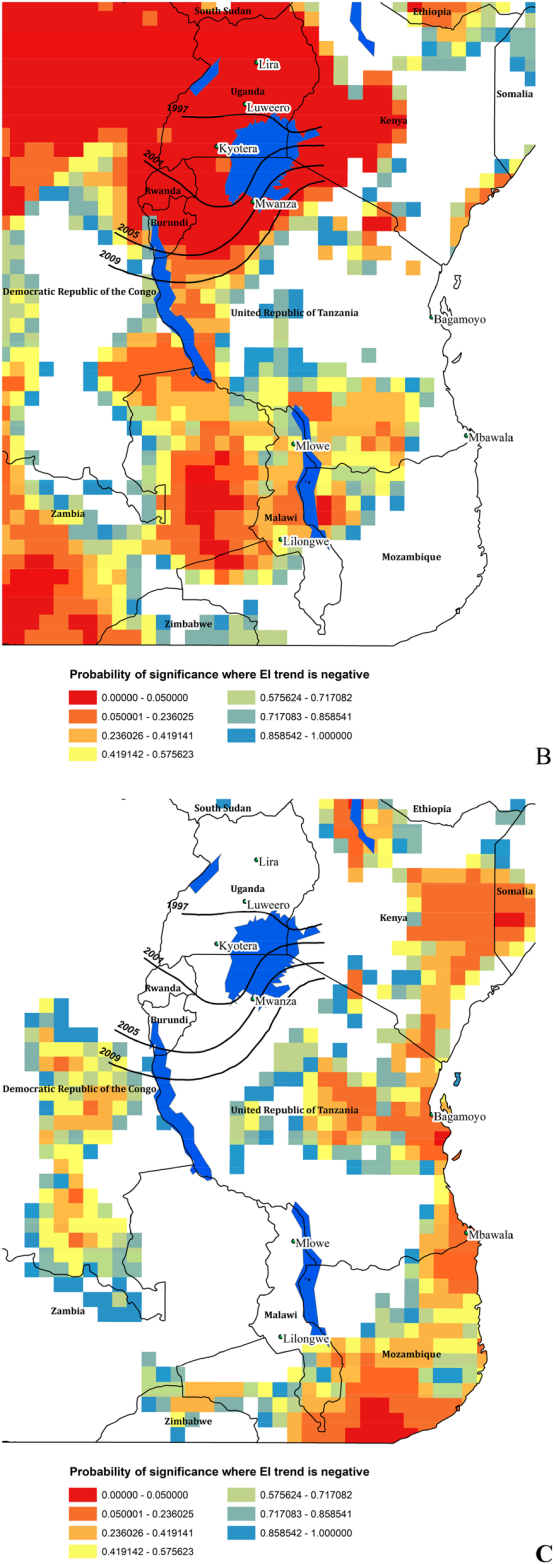
East and Central Africa showing changing climate suitability between 1979 and 2012 for *Bemisia tabaci* MEAM1 modelled using CLIMEX^53,64^ running the CRU time series climatic dataset^77^. (**A**) average change in Ecoclimatic Index, and the probability of significance of Student’s *t* statistic for a regression of the time-series within each cell of the average change in Ecoclimatic Index for (**B**) positive trend, and (**C**) for negative trend. Maps produced using ArcMap 10.6 (ESRI, Redlands, Ca., esri.com).

**Figure 7:**
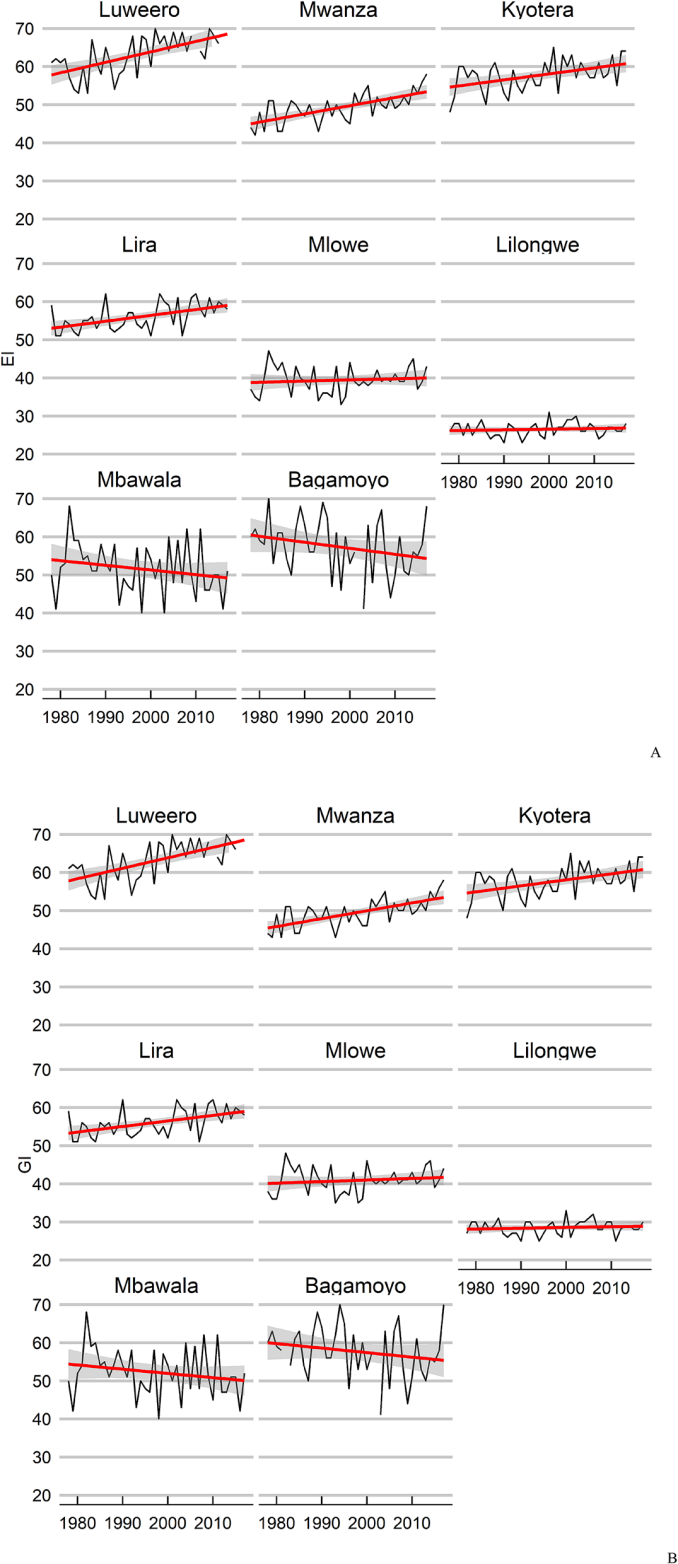

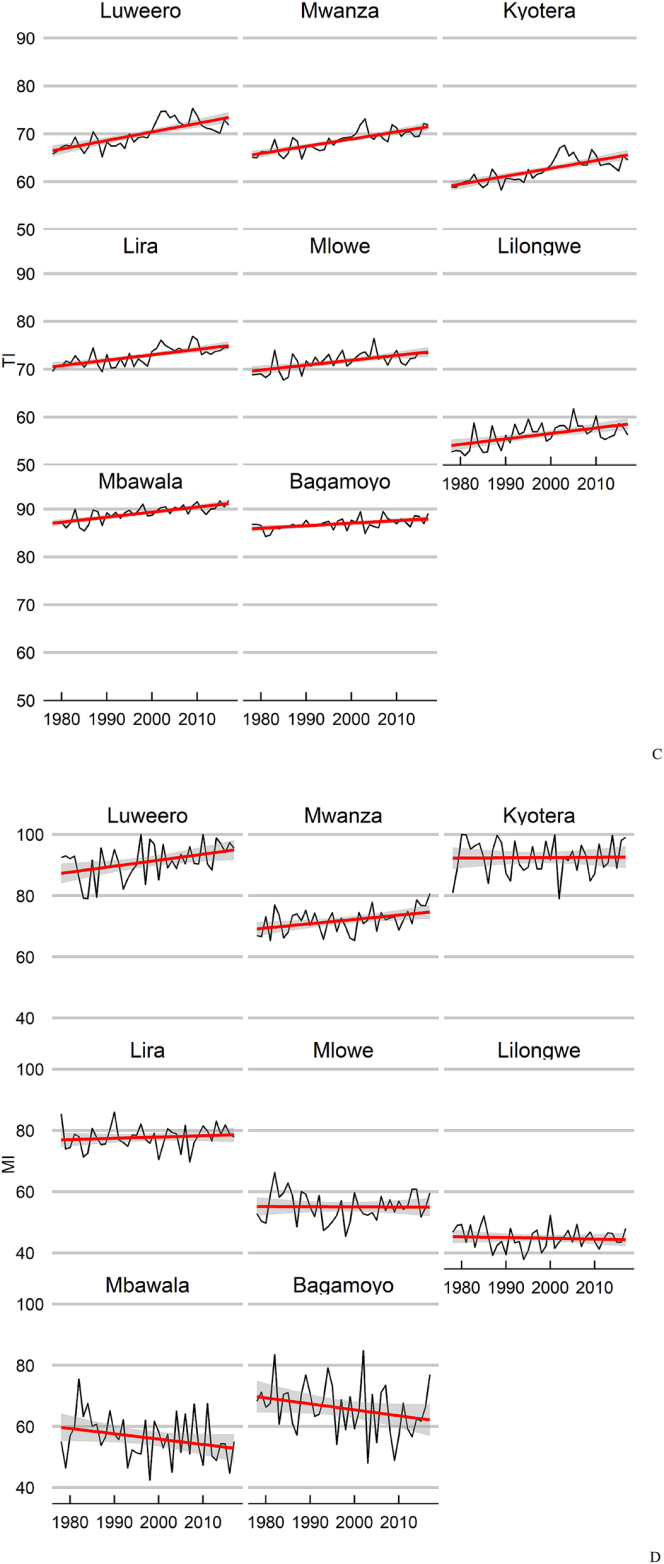
Time-series of annual climate suitability for *Bemisia tabaci* MEAM1 between 1989 and 2011 for selected locations in East Africa modelled using CLIMEX^53,64^ running the CRU time series dataset Mitchell^77^. (**A**) Ecoclimatic Index (EI), (**B**) Annual Growth Index (GI_A_), (**C**) Temperature Index (TI) and (**D**) Moisture Index (MI). The locations were selected to span a range of climatic trends spanning the three focal countries for the study (Uganda, Tanzania and Malawi). Locations are identified in Fig. 6.

There is considerable spatio-temporal variation in the modelled climate suitability for *B. tabaci* MEAM1 in eastern Africa at the inter-annual scale (Supplementary Figs. S2, S3). Despite this variability, there has been a significant trend in climate suitability throughout much of Eastern Africa during the 39 years from 1979 to 2017 (Fig. 6). For each cell there were 38 degrees of freedom. Throughout this period, there has been a slight decrease in climate suitability for *B. tabaci* in lower-lying coastal regions (Fig. 6a), though apart from an area in Mozambique, only a few isolated areas appear to have experienced a statistically significant decrease in suitability (Fig. 6c). Conversely, there has been an increase in suitability throughout a large portion of the area, which is most pronounced in the Democratic Republic of the Congo, Uganda, Rwanda, Burundi, and western parts of Tanzania and Kenya (Fig. 6a,b). These trends are apparent in both the EI and the GI_A_ results against a backdrop of interannual variation (Fig. 7a,b). Exploring the temporal data in more detail for selected locations reveals that most of the increase in suitability is due to increasing temperature suitability (Fig. 7c) and decreases in the EI are due to decreasing soil moisture suitability and drought stress (Fig. 7d). At Luweero and Mwanza, in the centre of the cassava disease epidemic zone, climatic conditions for both temperature and soil moisture improved through this period.

## Discussion

Both the field and laboratory studies support the application of the CLIMEX model originally fitted to development data and field distribution records for *B. tabaci* MEAM1 to examine the historical change in climate suitability for the *B. tabaci* SSA species. This lack of variation in the fitted model parameters between the different *B. tabaci* species is perhaps not so surprising. Members of the complex are often found to have sympatric ranges. *Bemisia tabaci* MED and MEAM1 frequently coexist^64,83^, and in East Africa the SSA taxa frequently co-occur^43^. It is likely that the non-climatic ecological factors such as host relations (e.g.,^83^) provide bases for niche differentiation within the *B. tabaci* species complex.

The Ugandan time-series data shows both a statistically consistent relationship between *B. tabaci* abundance and modelled climate suitability (Fig. 5), and an increasing suitability trend across time. The apparent increasing climate suitability for SSA *B. tabaci* in parts of East and Central Africa across the 39 years of the study (including the 13 years of the Ugandan dataset) accords with the reported increased prevalence of both *B. tabaci*^33^ and cassava diseases caused by viruses vectored by *B. tabaci*^26,27^. This sustained increase in climate suitability may help explain the increasing abundance of SSA *B. tabaci*^27^, in combination with increased use of cassava varieties that support *B. tabaci* species, and changes to cropping systems that provide additional host plant resources^40^. An increase in suitability across time was most obvious around the lake zone of Southern Uganda and Northern Tanzania, and west into Rwanda. This matches a more than five-fold increase in observed abundance of *B. tabaci* in this area between 1994 and 2009^33^. In contrast, parts of Malawi have seen little change in the climate suitability during this historical time period. Some sites such as Lira in Northern Uganda and coastal Tanzania have seen a slight decrease in suitability for *B. tabaci;* between 1994 and 2009, Jeremiah, et al.^33^ observed a three-fold decrease in abundance at coastal Tanzanian sites, which accords with the model results for Mbawala and Bagmoyo (Fig. 7).

In order to attribute these observed changes to anthropogenic climate change, the observed changes need to satisfy the following conditions:

*…unlikely to be entirely due to internal variability; consistent with the estimated responses to the given combination of anthropogenic and natural forcing; and not consistent with alternative, physically plausible explanations of recent climate change that exclude important elements of the given combination of forcings.”*^84^^:700^.

As observed in Rosenzweig et al.^18^, attributing climatic changes to natural systems requires special treatment through ‘joint attribution’. It seems clear that the recent reports of increased abundance of whiteflies and cassava diseases are not due simply to natural variation (internal variability). Cassava has been grown in the region for a long time prior to the recent reports of disease pandemics, and there have been a range of native whiteflies present in the region^43^. By using the CLIMEX model of *B. tabaci* MEAM1^64^, we have satisfied the second and third criteria for climate change attribution. The CLIMEX model was fitted using long-term average historical climate data and experimental data on MEAM1 response to climate variables (cross-checked with SSA responses), thus satisfying the plausibility criterion. When we applied the time-series climate data (*natural forcing*), the resulting pattern of statistically significant changes in climate suitability matched the observed patterns of changes in whitefly abundance and cassava disease. The trend was clearly sustained throughout the 39 years of the study, and was apparent despite the substantial inter-annual variability in climate suitability; further supporting the notion that it was not due to internal variability in the system, nor due to shorter-term variability in climate (e.g. inter-decadal)^15^. The null hypothesis for climate change is in-effect the baseline CLIMEX model for *B. tabaci* MEAM1, applied to the historical climate dataset centred on 1995. By drawing upon a process-oriented model such as CLIMEX in this manner, we are essentially utilizing a variant of the ‘end-to-end’ method of climate change attribution^18^.

In attributing the *B. tabaci* abundance and cassava disease changes to climatic changes we also need to consider other potential explanations and confounding effects. As observed by McQuaid et al.^85^, the movement of diseased cassava material can play an important role in the spread of a disease, especially over longer distances. However, the subsequent maintenance of an epidemic relies upon either a local cycling of the disease or constant re-introduction of diseased plant material. While farmers may spread diseased material locally, this is likely a small effect. Conversely, there is compelling evidence that *B. tabaci* plays a strong role in spreading the viruses within the landscape and maintaining epidemics^85,86^, so we can be confident that while the spread of diseased cassava cuttings into the epidemic zones was a factor in the spread of the disease^71^, the observed epidemic was most likely driven by *B. tabaci* dynamics and abundance.

The statistically significant trend of improving climate suitability for *B. tabaci* in the cassava disease pandemic area in East and Central Africa, centred mainly on Uganda appears to be mostly driven by increasing temperatures (Fig. 7c). At some sites at the centre of the recent cassava disease epidemics (e.g. Luweero, Uganda and Mwanza, Tanzania), decreasing rainfall also contributed to the increasing favourability for *B. tabaci* via the increase in the Moisture Index (Fig. 7d). This accords with the general perception of *B. tabaci* having a preference for hot, dry conditions^87^, and references therein. The preference for dry conditions is at least partly due to the susceptibility of nymphs to being dislodged from leaves by raindrops, a fact that has led to overhead irrigation being used as a means of suppressing *B. tabaci*^82,88^.

Where there were trends toward decreased suitability (e.g., in sub-coastal Tanzania and northern Kenya, Fig. 6a,c) the decreases also appear to be associated with decreasing rainfall. However, these predominantly rangeland areas were already poorly suited for cropping due to inadequate rainfall and became even drier and less suitable for *B. tabaci* during the period of this study. In East Africa, the trends in increasing climatic suitability for *B. tabaci* appear to be correlated with the increase in reported prevalence of SSA *B. tabaci* species and the rapid spread of *B. tabaci*-transmitted cassava diseases^26,28,30^. This result perhaps provides the first evidence for predicted effects of climate change contributing to the emergence of infectious plant diseases^89,90^ vectored by an arthropod^91,92^. This result then begs the question of how to use climate change scenarios to assess the potential extent of these viral epidemics, though this is beyond the scope of the present study.

Given the apparent increasing climate suitability trend for *B. tabaci* in parts of East and Southern Africa, we might expect that there could be more generations of *B. tabaci* and greater survival rates, and hence abundance. African Cassava Mosaic Virus (ACMV) prevalence is related to the density of whiteflies^93^, so we might reasonably expect that the increasing climatic suitability for *B. tabaci* could translate into greater disease prevalence. While Jeremiah et al.^33^ did not find a positive correlation between *B. tabaci* abundance and incidence of Cassava Brown Streak Disease (CBSD), based on epidemiological theory we might also expect that increasing *B. tabaci* density would have a proportionally greater effect on the levels of cassava diseases with relatively poor transmission rates (e.g. CBSD) compared with those with high transmission rates (e.g. CMD or ACMV) because the latter would saturate at lower density levels of *B. tabaci*. Logically, because transmission rates are observed on a per-insect basis, higher insect density results in higher plant infection rates, up to the point where the availability of uninfected hosts becomes limiting.

The sustained trend in climatic suitability for *B. tabaci* in parts of East and Central Africa suggests that a similarly sustained effort will be required to develop and maintain cassava varieties that are resistant to current and emerging strains of cassava diseases. Otherwise, the contribution of cassava production toward African and global food security will likely be compromised. Clearly, such plant breeding initiatives need to be paralleled with efforts to control *B. tabaci* using biological and cultural control methods. Methods such as those employed by Pardey et al.^94^ to estimate the economically appropriate amount to invest in perpetuity in developing and maintaining resistance to wheat stem rust (*Puccinia graminis*) might be applied to the protection of cassava production from arboviruses. Instead of framing this problem as a battle to be won—a project with a start and end date—it can be framed as a perpetual investment stream to develop cassava varieties that are resistant to the contemporary challenges from the evolving patho-system.

The newly available Compare Locations/Years tool in CLIMEX Version 4, coupled with gridded time series climatic data allowed us to explore the effects of climate variability on this important agricultural pest system. It enabled us to look not only at the resultant trends, but also to identify which components of climate were driving these trends. Linking climate dynamics to a process-oriented niche model is potentially an important mechanism for exploring trends in global change biology. The process-oriented nature of CLIMEX, combined with time series climatic data, lends it to serving a useful role in climate change attribution in many biological systems.

Our modelling identified that East and Southern Africa includes areas that are highly climatically suitable for *B. tabaci* SSA species*,* and that this suitability has been increasing for many years in certain regions, highlighting an additional potential biosecurity and food security threat for the region. At present, the native *B. tabaci* SSA species already pose a significant threat to cassava production, and there are other related whitefly species also present in cassava fields. The role that native African whitefly species play in terms of transmission of a large diversity of cassava viruses is sometimes unclear. Africa’s notoriously porous land borders^95,96^ make it difficult to control the spread of invasive species and emerging infectious diseases. West African cassava production is greater than that in East Africa. If Cassava brown streak virus disease or Ugandan cassava brown streak virus were to be introduced to West Africa, for example via the movement of infected cassava cuttings, the threat to cassava production there could be even greater than that observed in East Africa if a suitable whitefly vector is either already present, or invades that area. Clarifying the ability of West African whiteflies to transmit CBSV and UCBSV, may help define a range of strategies to manage these disease risks to cassava production in this region.

## Supplementary Information

Supplementary Information

## References

[1] Kriticos DJ, Sutherst RW, Brown JR, Adkins SA, Maywald GF (2003). Climate change and the potential distribution of an invasive alien plant: *Acacia nilotica* ssp. indica in Australia. J. Appl. Ecol..

[2] Sutherst RW, Canadell JG, Pataki DE, Pitelka LF (2007). Pests under global change—meeting your future landlords?. Terrestrial Ecosystems in a Changing World.

[3] Sutherst, R.W., Arthropods as disease vectors in a changing environment. In *Ciba Foundation Symposium 175—Environmental Change and Human Health* (Wiley, 2007), pp. 124–145. 10.1002/9780470514436.ch8 8222987

[4] Vogl G (2008). Modelling the spread of ragweed: Effects of habitat, climate change and diffusion. Eur. Phys. J. Spec. Top..

[5] Scherm, H., Climate change: can we predict the impacts on plant pathology and pest management? Presented at the Annual Meeting of the Canadian-Phytopathological-Society, Montreal, Canada, 2003 (unpublished), pp. 267–273.

[6] Kocmankova E (2011). Estimating the impact of climate change on the occurrence of selected pests at a high spatial resolution: A novel approach. J. Agric. Sci..

[7] Mardulyn P (2013). Climate change and the spread of vector-borne diseases: Using approximate Bayesian computation to compare invasion scenarios for the bluetongue virus vector *Culicoides imicola* in Italy. Mol. Ecol..

[8] Ziter C, Robinson EA, Newman JA (2012). Climate change and voltinism in Californian insect pest species: Sensitivity to location, scenario and climate model choice. Glob. Change Biol..

[9] Estay SA, Lima M, Labra FA (2009). Predicting insect pest status under climate change scenarios: Combining experimental data and population dynamics modelling. J. Appl. Entomol..

[10] Parmesan C (1999). Poleward shifts in geographical ranges of butterfly species associated with regional warming. Nature.

[11] Parmesan C (2005). Empirical perspectives on species borders: From traditional biogeography to global change. Oikos.

[12] Kerdelhue C (2009). Quaternary history and contemporary patterns in a currently expanding species. BMC Evol. Biol..

[13] Battisti A (2005). Expansion of geographic range in the pine processionary moth caused by increased winter temperature. Ecol. Appl..

[14] Rahmstorf S (2007). Recent climate observations compared to projections. Science.

[15] Mann ME, Lees JM (1996). Robust estimation of background noise and signal detection in climatic time series. Clim. Change.

[16] Parmesan C (2006). Ecological and evolutionary responses to recent climate change. Annu. Rev. Ecol. Evol. Syst..

[17] Bloomfield P, Nychka D (1992). Climate spectra and detecting climate change. Clim. Change.

[18] Rosenzweig C (2008). Attributing physical and biological impacts to anthropogenic climate change. Nature.

[19] Sutherst RW (2011). Adapting to crop pest and pathogen risks under a changing climate. Wiley Interdiscip. Rev. Clim. Change.

[20] FAOSTAT (2015). Crop Production.

[21] Nweke FI (2004). New Challenges in the Cassava Transformation in Nigeria and Ghana.

[22] Godfray HCJ (2010). Food security: The challenge of feeding 9 billion people. Science.

[23] El-Sharkawy MA (2004). Cassava biology and physiology. Plant Mol. Biol..

[24] Jarvis A, Ramirez-Villegas J, Herrera Campo B, Navarro-Racines C (2012). Is Cassava the answer to African climate change adaptation?. Trop. Plant Biol..

[25] Howeler R, Lutaladio N, Thomas G (2013). Save and Grow: Cassava. A Guide to Sustainable Production Intensification.

[26] Alicai T (2007). Re-emergence of Cassava Brown Streak Disease in Uganda. Plant Dis..

[27] Colvin J, Omongo CA, Maruthi MN, Otim-Nape GW, Thresh JM (2004). Dual begomovirus infections and high *Bemisia tabaci* populations: Two factors driving the spread of a cassava mosaic disease pandemic. Plant. Pathol..

[28] Legg JP (2014). Spatio-temporal patterns of genetic change amongst populations of cassava *Bemisia tabaci* whiteflies driving virus pandemics in East and Central Africa. Virus Res..

[29] Thresh J (1997). African cassava mosaic virus disease: The magnitude of the problem. Afr. J. Root Tuber Crops.

[30] Tajebe LS (2014). Abundance, diversity and geographic distribution of cassava mosaic disease pandemic-associated *Bemisia tabaci* in Tanzania. J. Appl. Entomol..

[31] Ndunguru J (2015). Analyses of twelve new whole genome sequences of Cassava Brown Streak Viruses and Ugandan Cassava Brown Streak Viruses from East Africa: Diversity, supercomputing and evidence for further speciation. PLoS One.

[32] Basavaprabhu LP, Legg JP, Kanju E, Fauquet CM (2015). Cassava brown streak disease: A threat to food security in Africa. J. Gen. Virol..

[33] Jeremiah SC (2015). The dynamics and environmental influence on interactions between Cassava Brown Streak Disease and the whitefly,. Phytopathology.

[34] Legg J, Owor B, Sseruwagi P, Ndunguru J (2006). Cassava mosaic virus disease in East and Central Africa: Epidemiology and management of a regional pandemic. Adv. Virus Res..

[35] FAO, Cassava Diseases in central, eastern and southern Africa: Strategic programme framework 2010–2015. (2009).

[36] Zhou X (1997). Evidence that DNA-A of a geminivirus associated with severe cassava mosaic disease in Uganda has arisen by interspecific recombination. J. Gen. Virol..

[37] Legg JP, French R, Rogan D, Okao-Okuja G, Brown JK (2002). A distinct *Bemisia tabaci* (Gennadius) (Hemiptera: Sternorrhyncha: Aleyrodidae) genotype cluster is associated with the epidemic of severe cassava mosaic virus disease in Uganda. Mol. Ecol..

[38] Garrett, K.A., Thomas-Sharma, S., Forbes, G.A., & Nopsa, J.H., Climate change and plant pathogen invasions. In *Invasive Species and Global Climate Change* (eds Ziska, L. H., Dukes, J. S.) 22 (2014).

[39] Tay WT (2017). The trouble with MEAM2: Implications of pseudogenes on species delimitation in the globally invasive *Bemisia tabaci* (Hemiptera: Aleyrodidae) cryptic species complex. Genome Biol. Evol..

[40] Macfadyen S (2018). Cassava whitefly, Bemisia tabaci (Gennadius) (Hemiptera: Aleyrodidae) in East African farming landscapes: A review of the factors determining abundance. Bull. Entomol. Res..

[41] Sseruwagi P, Sserubombwe W, Legg J, Ndunguru J, Thresh J (2004). Methods of surveying the incidence and severity of cassava mosaic disease and whitefly vector populations on cassava in Africa: A review. Virus Res..

[42] Boykin LM (2018). Review and guide to a future naming system of African *Bemisia tabaci* species. Syst. Entomol..

[43] Mugerwa H (2018). African ancestry of New World, *Bemisia tabaci*-whitefly species. Sci. Rep..

[44] Boykin LM, Armstrong KF, Kubatko L, De Barro PJ (2011). Species delimitation and global biosecurity. Evol. Bioinform..

[45] De Barro PJ, Liu S-S, Boykin LM, Dinsdale AB (2011). *Bemisia tabaci*: A statement of species status. Annu. Rev. Entomol..

[46] Boykin LM (2014). *Bemisia tabaci* nomenclature: Lessons learned. Pest Manag. Sci..

[47] Kalyebi A (2018). African cassava whitefly, *Bemisia tabaci*, cassava colonization preferences and control implications. PLoS One.

[48] Herrera Campo B, Hyman G, Bellotti A (2011). Threats to cassava production: Known and potential geographic distribution of four key biotic constraints. Food Secur..

[49] Webber BL (2011). Modelling horses for novel climate courses: Insights from projecting potential distributions of native and alien Australian acacias with correlative and mechanistic models. Div. Distrib..

[50] Sutherst RW, Bourne AS (2009). Modelling non-equilibrium distributions of invasive species: A tale of two modelling paradigms. Biol. Invas..

[51] Ramos RS, Kumar L, Shabani F, Picanço MC (2018). Mapping global risk levels of *Bemisia tabaci* in areas of suitability for open field tomato cultivation under current and future climates. PLoS One.

[52] Lobo JM, Jiménez-Valverde A, Real R (2008). AUC: A misleading measure of the performance of predictive distribution models. Glob. Ecol. Biogeogr..

[53] Kriticos DJ (2015). CLIMEX Version 4: Exploring the Effects of Climate on Plants, Animals and Diseases.

[54] Sutherst RW, Maywald GF (1985). A computerised system for matching climates in ecology. Agric. Ecosyst. Environ..

[55] Yonow T, Hattingh V, de Villiers M (2013). CLIMEX modelling of the potential global distribution of the citrus black spot disease caused by *Guignardia citricarpa* and the risk posed to Europe. Crop Prot..

[56] Ireland KB, Hardy GESJ, Kriticos DJ (2013). Combining inferential and deductive approaches to estimate the potential geographical range of the invasive plant pathogen, *Phytophthora ramorum*. PLoS One.

[57] Kriticos DJ (2017). The potential global distribution of the brown marmorated stink bug, *Halyomorpha halys*, a critical threat to plant biosecurity. J. Pest Sci..

[58] Macfadyen S, Kriticos DJ (2012). Modelling the geographical range of a species with a variable life-history. PLoS One.

[59] Yonow T, Sutherst RW (1998). The geographical distribution of the Queensland fruit fly, *Bactrocera* (*Dacus*) *tryoni*, in relation to climate. Aust. J. Agric. Res..

[60] De Villiers M (2016). The potential distribution of *Bactrocera dorsalis*: Considering phenology and irrigation patterns. Bull. Entomol. Res..

[61] De Villiers M, Hattingh V, Kriticos DJ (2012). Combining field phenological observations with distribution data to model the potential range distribution of the fruit fly *Ceratitis rosa* Karsch (Diptera: Tephritidae). Bull. Entomol. Res..

[62] Zalucki MP, Furlong MJ (2005). Forecasting *Helicoverpa* populations in Australia: A comparison of regression based models and a bio-climatic based modelling approach. Insect Sci..

[63] Zalucki MP, Van Klinken RD (2006). Predicting population dynamics of weed biological control agents: Science or gazing into crystal balls?. Aust. J. Entomol..

[64] Kriticos DJ, De Barro PJ, Yonow T, Ota N, Sutherst RW (2020). The potential geographical distribution and phenology of *Bemisia tabaci* Middle East Asia Minor 1, considering irrigation and glasshouse production. Bull. Entomol. Res..

[65] Kriticos DJ (2012). CliMond: Global high resolution historical and future scenario climate surfaces for bioclimatic modelling. Methods Ecol. Evol..

[66] Hutchinson, G.E., Presented at the Cold Spring Symposium on Quantitative Biology, Yale University, New Haven, Connecticutt, USA, 1957 (unpublished).

[67] Brown JH, Stevens GC, Kaufman DM (1996). The geographic range: Size, shape, boundaries, and internal structure. Annu. Rev. Ecol. Syst..

[68] Peterson AT, Soberon J, Pearson RG, Martinez-Meyer E (2011). Ecological Niches and Geographic Distributions.

[69] Davis AJ, Jenkinson LS, Lawton JH, Shorrocks B, Wood S (1998). Making mistakes when predicting shifts in species range in response to global warming. Nature.

[70] Carter RN, Prince SD (1981). Epidemic models used to explain biogeographical distribution limits. Nature.

[71] Alicai T (2019). Expansion of the cassava brown streak pandemic in Uganda revealed by annual field survey data for 2004 to 2017. Sci. Data.

[72] Macfadyen S (2020). Landscape factors and how they influence whitefly pests in cassava fields across East Africa. Landsc. Ecol..

[73] Shelford VE (1963). The Ecology of North America.

[74] Shelford VE (1918). A comparison of the responses of animals in gradients of environmental factors with particular reference to the method of reaction of representatives of the various groups from protozoa to mammals. Science.

[75] Shelford VE, Deere EO (1913). The reactions of certain animals to gradients of evaporating power of air: A study in experimental ecology. Biol. Bull..

[76] van der Ploeg RR, Böhm W, Kirkham MB (1999). On the origin of the theory of mineral nutrition of plants and the law of the minimum. Soil Sci. Soc. Am. J..

[77] Mitchell TD, Jones PD (2005). An improved method of constructing a database of monthly climate observations and associated high-resolution grids. Int. J. Climatol..

[78] New M, Hulme M, Jones P (2000). Representing twentieth-century space-time climate variability. Part II: Development of 1901–96 monthly grids of terrestrial surface climate. J. Clim..

[79] Sseruwagi P (2006). Colonization of non-cassava plant species by cassava whiteflies (*Bemisia tabaci*) in Uganda. Entomol. Exp. Appl..

[80] Otim-Nape G, Alicai T, Thresh J (2001). Changes in the incidence and severity of cassava mosaic virus disease, varietal diversity and cassava production in Uganda. Ann. Appl. Biol..

[81] R Core Team (2013). R: A Language and Environment for Statistical Computing.

[82] Castle S, Henneberry T, Toscano N (1996). Suppression of *Bemisia tabaci* (Homoptera: Aleyrodidae) infestations in cantaloupe and cotton with sprinkler irrigation. Crop Prot..

[83] Alemandri V (2015). Three members of the *Bemisia tabaci* (Hemiptera: Aleyrodidae) cryptic species complex occur sympatrically in Argentine horticultural crops. J. Econ. Entomol..

[84] Mitchell, J. *et al.*, Detection of climate change and attribution of causes in *IPCC 2001: Climate Change 2001. The Climate change Contribution of Working Group I to the Third Assessment Report of the Intergovemmental Panel on Climate Change*, edited by J Houghton *et al.* (2001), Vol. 159.

[85] McQuaid CF (2017). Spatial dynamics and control of a crop pathogen with mixed-mode transmission. PLoS Comput. Biol..

[86] Fauquet C, Fargette D (1990). African cassava mosaic virus: Etiology, epidemiology and control. Plant Dis..

[87] Aregbesola OZ, Legg JP, Sigsgaard L, Lund OS, Rapisarda C (2018). Potential impact of climate change on whiteflies and implications for the spread of vectored viruses. J. Pest. Sci..

[88] Hilje L, Costa HS, Stansly PA (2001). Cultural practices for managing Bemisia tabaci and associated viral diseases. Crop Prot..

[89] Anderson PK (2004). Emerging infectious diseases of plants: Pathogen pollution, climate change and agrotechnology drivers. Trends Ecol. Evol..

[90] Chakraborty S, Tiedemann AV, Teng PS (2000). Climate change: Potential impact on plant diseases. Environ. Pollut..

[91] Jones RA (2009). Plant virus emergence and evolution: Origins, new encounter scenarios, factors driving emergence, effects of changing world conditions, and prospects for control. Virus Res..

[92] Canto T, Aranda MA, Fereres A (2009). Climate change effects on physiology and population processes of hosts and vectors that influence the spread of hemipteran-borne plant viruses. Glob. Change Biol..

[93] Fargette D, Jeger M, Fauquet C, Fishpool L (1994). Analysis of temporal disease progress of African cassava mosaic virus. Phytopathology.

[94] Pardey PG (2013). Right-sizing stem rust research. Science.

[95] Dodson B (2000). Porous borders: Gender and migration in Southern Africa. S. Afr. Geogr. J..

[96] Ikome, F.N., Africa’s international borders as potential sources of conflict and future threats to peace and security (2012).

