# Supplementary material for: Improving climate suitability for Bemisia tabaci in East Africa is correlated with increased prevalence of whiteflies and cassava diseases
Source: Sci Rep. 2020 Dec 16;10:22049. doi: 10.1038/s41598-020-79149-6 (PMC7744558; doi:10.1038/s41598-020-79149-6)
Supplement: Supplementary file 1 — Supplementary Information [file 41598_2020_79149_MOESM1_ESM.docx]

**Improving climate suitability for *Bemisia tabaci* in East Africa is correlated with increased prevalence of whiteflies and cassava diseases: Supplementary Information**

Darren J. Kriticos, Ross E. Darnell, Tania Yonow, Noboru Ota, Robert W. Sutherst, Hazel Parry, Habibu Mugerwa, M.N. Maruthi, Susan E. Seal, John Colvin, Sarina Macfadyen, Andrew Kalyebi, Andrew Hulthen and Paul J. De Barro


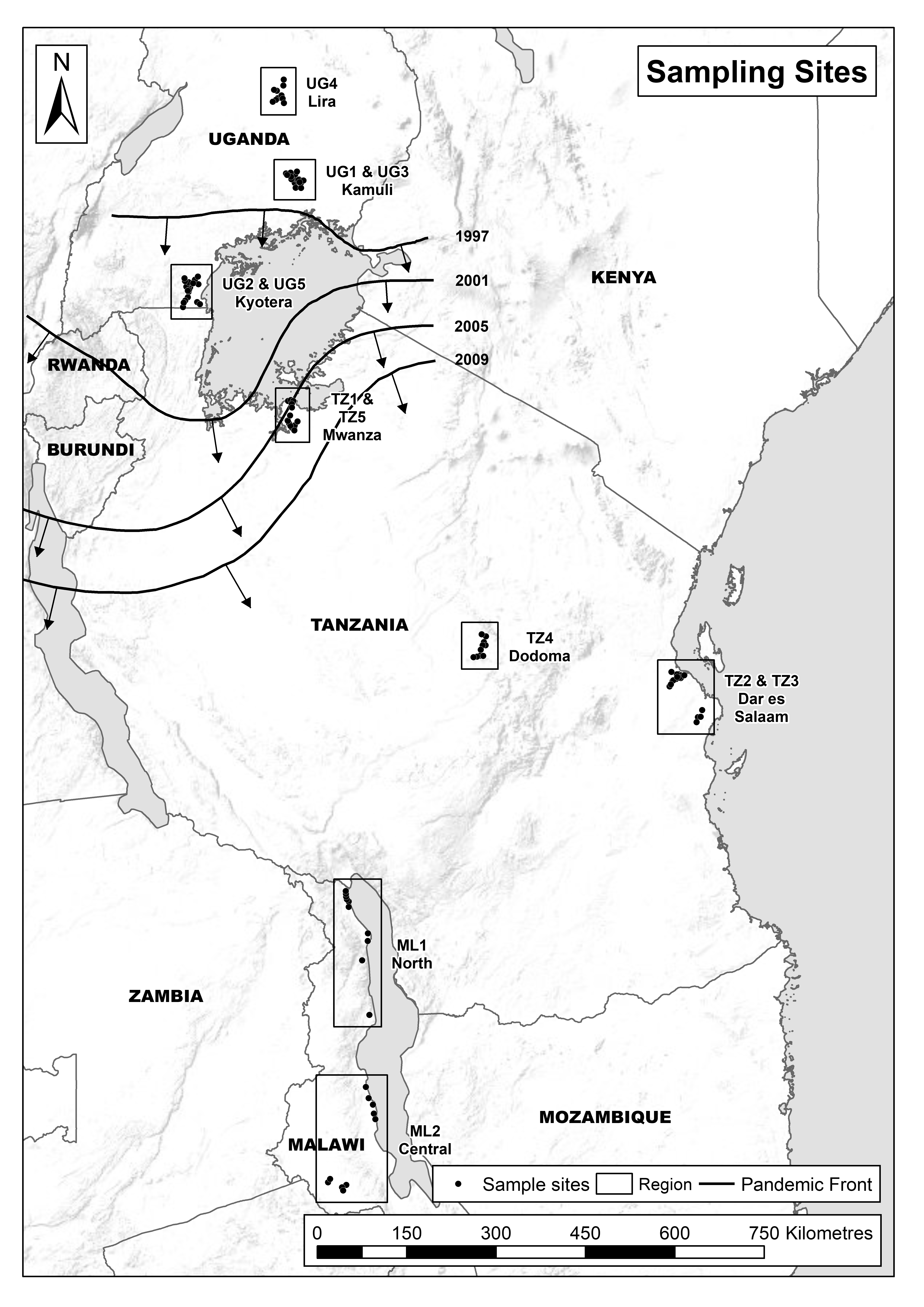


**Figure S1. Distribution of study sites for *Bemisia tabaci* and cassava disease surveys. Produced using ArcMap 10.6 (ESRI, Redlands, Ca., esri.com)**


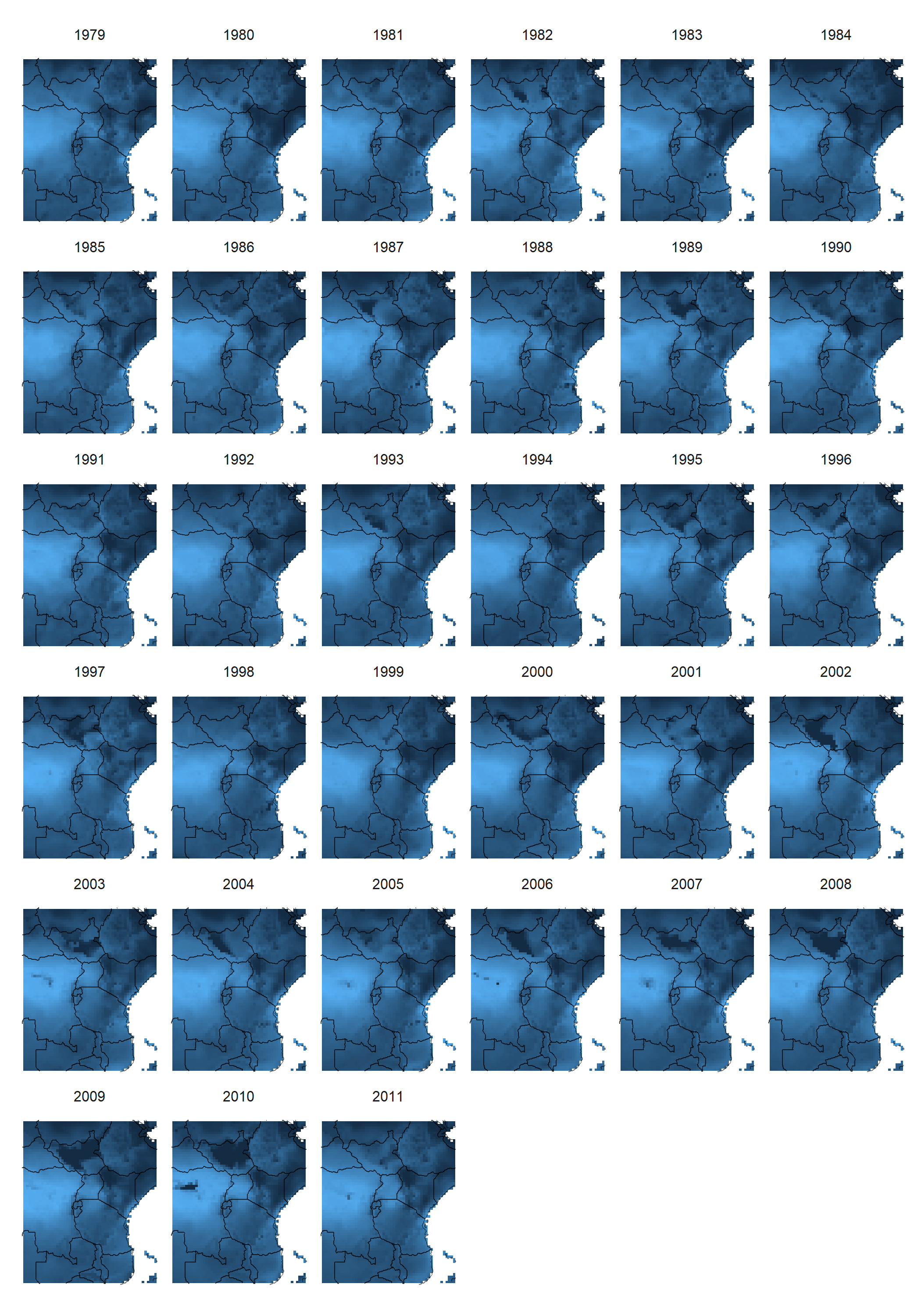


Figure S2. Climate suitability for persistence as indicated using the CLIMEX Ecoclimatic Index. Dark areas are unsuitable. Produced using R version 3.5.1 and R Studio version 1.0.143.


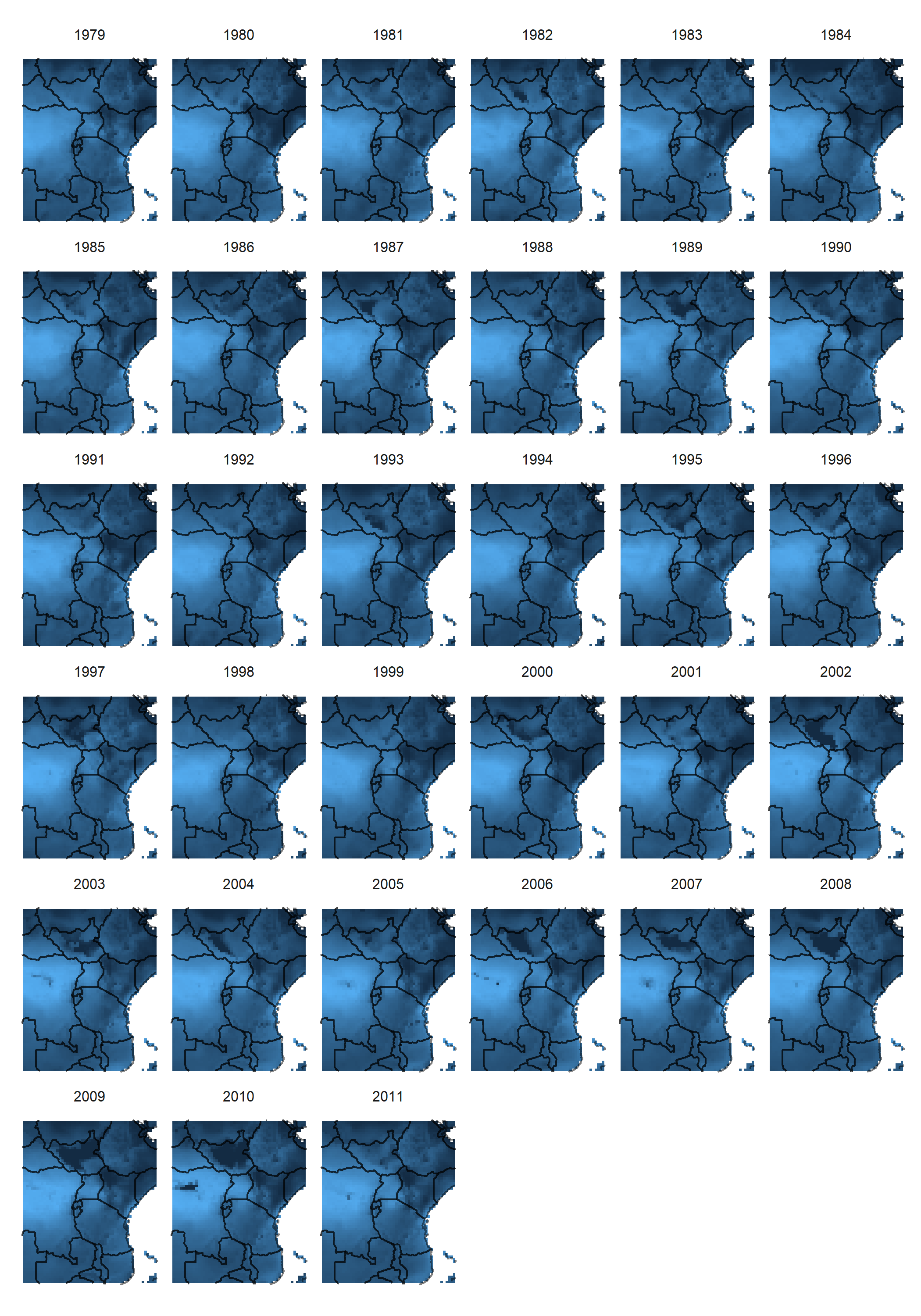


Figure S3. Climate suitability for population growth as indicated using the CLIMEX Annual Growth Index (GI_A_). Dark areas are unsuitable for growth. Produced using R version 3.5.1 and R Studio version 1.0.143.
